# Specific imaging of bacterial infection: a translational approach using positron emission tomography and gallium-68-labeled maltohexaose

**DOI:** 10.7150/thno.125904

**Published:** 2026-03-30

**Authors:** Sophie M. Wegener, Desiree Weiberg, James T. Thackeray, Christoph P. Czerner, Jasmin S. Hanke, Jan D. Schmitto, Geerd-Jürgen Meyer, Jens P. Bankstahl, Arjang Ruhparwar, Frank M. Bengel, Tobias L. Ross

**Affiliations:** 1 Department of Nuclear Medicine, Hannover Medical School; Carl-Neuberg-Str. 1, 30625 Hannover, Germany.; 2 Department of Cardiac, Thoracic, Transplantation and Vascular Surgery, Hannover Medical School, Carl-Neuberg-Str. 1, 30625 Hannover, Germany.

**Keywords:** positron emission tomography, molecular imaging, infection, maltodextrins, bacteria-targeted imaging

## Abstract

**Rationale:**

Direct visualization of the presence and biological activity of bacterial pathogens in infectious foci may be of considerable value for individualized infection medicine. We hypothesized that a positron-emitting maltodextrin radiotracer [^68^Ga]Gallium-DOTA-maltohexaose ([^68^Ga]Ga-DMALTO) could sensitively and translationally distinguish between bacterial infection and aseptic inflammation in a clinical application.

**Methods:**

A new radiosynthesis of [^68^Ga]Ga-DMALTO was established and transferred to an automated good manufacturing practice-compatible automated setting. [^68^Ga]Ga-DMALTO was analyzed *in vitro* in bacterial strains and macrophages followed by *in vivo* positron emission tomography (PET) in healthy/infected/inflamed mouse models. Finally, it was examined in an early clinical application in patients with left-ventricular assist device infection, requiring precise localization of infection foci.

**Results:**

A robust, high-yield radiosynthesis of [^68^Ga]Ga-DMALTO was established and *in vitro*-assays confirmed specific uptake in bacteria with no uptake in macrophages. In mouse models, [^68^Ga]Ga-DMALTO was rapidly cleared via renal excretion with minimal background in healthy tissue. In the presence of Gram-negative (*Escherichia coli*) or Gram-positive (*Bacillus subtilis*) bacterial infection the [^68^Ga]Ga-DMALTO signal was specifically located in infected hindlimbs, which was not observed in sterile inflammation models. By contrast, the standard clinical tracer 2-[^18^F]Fluoro-2-deoxy-D-glucose ([^18^F]FDG) did not distinguish between infection and aseptic inflammation. The clinical application of [^68^Ga]Ga-DMALTO in patients with left-ventricular assist device infection confirmed renal clearance and low background, and demonstrated specific accumulation in proven infectious foci along the driveline path. In comparison with [^18^F]FDG, focal [^68^Ga]Ga-DMALTO uptake was reproducible but less intense, supporting a complementary role for the *in vivo* imaging of viable bacterial burden and host inflammatory responses.

**Conclusions:**

The hypothesis-generating results of our work lay the foundation for future studies determining the specific benefit of bacteria-targeted *in vivo* imaging for clinical decision making and anti-infectious therapy guidance.

## Introduction

In complex clinical situations such as fever of unknown origin 1 or suspected infection of cardiovascular or other implants 2, standard approaches such as clinical examination, blood testing, and morphologic imaging have limited diagnostic certainty. Radionuclide-based molecular imaging can localize sites of inflammation and provide functional readouts of host immune cell recruitment and metabolism. Established probes such as technetium-99m- or indium-111-labeled autologous white blood cells for single photon emission computed tomography (SPECT), as well as [^18^F]FDG for positron emission tomography (PET), image inflammatory processes by targeting activated immune cells and metabolic activity, rather than the infectious agent itself 3. Consequently, the resulting imaging signal may not reliably distinguish bacterial, viral, and other pathogen-driven infections from aseptic inflammation associated with non-infectious tissue injury and repair, such as after surgical procedures. Given the increasing incidence and complexity of bacterial infections, there is an unmet clinical need for specific molecular imaging agents for bacteria 4. An imaging signal that directly identifies the infection location and bacterial burden may compliment or replace techniques targeting the inflammatory host response, thereby refining therapeutic decision-making.

Specific targeting of infected lesions can be based on peptides 5, 6, antibodies 7, macrophage targeting 8, 9, or targeting the pathogens themselves with small molecules 10-12. Maltodextrins are a major energy source for bacteria and they are internalized through the bacteria-specific maltodextrin-transporter pathway, which is absent from mammalian cells. Accordingly, radiolabeled maltodextrins may be particularly suitable probes for specific imaging of bacteria. Several experimental studies suggested that radiolabeled maltohexaose is a suitable compound for imaging of bacteria using multiple modalities, but clinical translation has not yet been established 13-15.

We hypothesized that radiolabeled maltohexaose is the perfect translational agent to bridge the clinical gap of bacteria-targeted *in vivo* imaging in individualized infection medicine. It would provide a quantitative and selective indication of bacterial infection and higher sensitivity than conventional imaging of the immune response. We established the radiosynthesis of [^68^Ga]Ga-labeled DOTA-maltohexaose ([^68^Ga]Ga-DMALTO), and tested tracer specificity for bacteria initially *in vitro* and in *in vivo* mouse models. Next, a good manufacturing practice (GMP)-compliant procedure was developed for human application, and subsequently used in the clinical setting of patients with left ventricular assist device (LVAD) infection 16.

## Materials and Methods

**Study design:** This project was designed as a translational effort, integrating *in vitro* studies, *in vivo* experimental animal work, and early clinical investigations.

*In vitro* work included the development of tracer synthesis and evaluation of the radiotracer in bacterial and mammalian cell cultures. All *in vitro* experiments were performed in triplicate unless otherwise specified.

Experimental animal studies were performed in representative models of hindlimb infection and aseptic inflammation with unaffected contralateral hindlimbs serving as internal controls. The study design was exploratory, and owing to the novel nature of the tested compound, no formal power calculation was performed. Sample sizes ranged from n = 3 - 10 per experimental group, as specified in the respective sections, and were based on common practice in prior work from our laboratory and other using small animal PET imaging of novel radiopharmaceuticals.

Clinical application was observational in an LVAD patient group where clinical PET imaging is indicated and where the German Pharmaceutical Act specifically enables clinical application of novel compounds. An exploratory analysis of the patient data was conducted using methodologies analogous to those employed in the experimental animal studies.

**Materials:** Chemicals were purchased from Sigma-Aldrich (St. Louis, USA), Dako (Agilent Technologies, CA, USA) or Merck Millipore (Darmstadt, Germany). Propargyl-DOTA-tris(tBu)ester was purchased from CheMatech (Dijon, France). Strata-X 33µ polymeric cartridges were purchased from Phenomenex (Aschaffenburg, Germany) and Microsep Advance Centrifugal Device (0.2 μm) was from Pall Life Science (Port Washington, NY, USA).

**Synthesis of DOTA-conjugated maltohexaose:** The synthesis of DOTA-maltohexaose was performed by copper(I)-catalyzed cycloaddition between an azide-functionalized maltohexaose (synthesized in a four-step method as described previously 17) and propargyl-DOTA-tris(tBu)ester 18, 19. A solution of CuSO_4_ (7.48 μmol) and sodium ascorbate (15.00 μmol) was dropped into a solution of azide-functionalized maltohexaose (2.67 μmol) and propargyl-DOTA-tris(tBu)ester (3.75 µmol) in ^tert^BuOH:water (1:1). After complete reaction, purification was performed on a Strata-X cartridge followed by precipitation of CuS and complete deprotection of DOTA-maltohexaose.

**Radiochemistry of [^68^Ga]Ga-DMALTO:** Labeling was performed using the eluate from an Obninsk [^68^Ge]Ge/[^68^Ga]Ga generator from Eckert & Ziegler (Berlin, Germany). The [^68^Ge]Ge/[^68^Ga]Ga generator was eluted with 0.1 M HCl and purified and concentrated on a strong anion exchange resin as described previously 20. Labeling of DOTA-maltohexaose was achieved in 1.5M HEPES buffer at 100 °C for 10 min. The obtained [^68^Ga]Ga-DMALTO ([^68^Ga]Ga-DOTA-maltohexaose) was used without any further purification. Quality control was achieved with radio-thin layer chromatography (TLC) [aluminum TLC plates, NH_2_-modified silica gel coated from Merck (Darmstadt, Germany) with citrate buffer as mobile phase, R_f_ = 1]. Radio-TLC and metabolism studies were validated with additional radio-high performance liquid chromatography (HPLC). A NUCLEODUR NH_2_-RP HPLC column (Macherey-Nagel, Düren, Germany) was used with a gradient of 50 - 100% citrate buffer and 50 - 0% acetonitrile for 0 - 10 min and 100% citrate buffer for 10 - 13 min with a retention time of 4.5 min for [^68^Ga]Ga-DMALTO. The early peak between 0 - 1 minute is the reference control peak, which comes from a first activity detector pass of the full sample without any retention on the HPLC column. The subsequent time course then reflects radioactivity after retention on the HPLC column.

**Animal models:** All animal experiments were conducted with the approval of the local authorities (approval no. 33.12-42502-04-15/1824), and in accordance with the European and German Animal Welfare Law. Sixty-eight male C57Bl/6N mice from Charles River (Germany) with a body weight of 26.8 ± 2.9 g were housed with a 14 h/10 h light/dark cycle with standard laboratory diet and water ad libitum. Animals were separated in three subgroups: healthy (no treatment), infected, and inflamed animals.

Infected hindlimb animal model: Bacterial strains *Escherichia coli* K12 (MG1655, ATCC 47076) and *Bacillus subtilis* (ATCC 23857) were grown in Difco LB Broth, Miller (Luria-Bertani) from BD (New Jersey, USA) overnight shaking at 37 °C. Bacterial strains were chosen to cover Gram-positive and -negative strains, while staying in Biosafety Level 1 to comply with our laboratory specifications. Bacteria were then subcultured into fresh LB and grown until log phase (A_600_ = 0.5). Mice were anesthetized by isoflurane and 30 µL of a bacteria suspension [10^5^ - 10^7^ colony forming units (CFU) in saline] was injected into the left *gastrocnemius* 14, 17. As a negative control, 30 µL of saline was injected into the right *gastrocnemius*.

Inflamed hindlimb animal model: Mice were anesthetized by isoflurane and 30 µL of lipopolysaccharide (LPS, Escherichia coli 0111:B4, Sigma-Aldrich, St. Louis, USA) in saline (0.9 mg/mL) was injected in the left *gastrocnemius* 17. As a negative control, 30 µL saline was injected in the right *gastrocnemius*.

**Small animal PET/CT imaging:** Under isoflurane anesthesia, mice were positioned on a mouse bed with stretched and fixed hind legs. Radiotracers (10.8 ± 2.0 MBq [^68^Ga]Ga-DMALTO or 8.9 ± 1.1 MBq [^18^F]FDG) were administered via the lateral tail vein. Dynamic PET was acquired over 60 min and static PET was acquired 50 - 60 min after radiotracer application on a Siemens Inveon small animal PET/CT (Knoxville, TN, USA). Dynamic PET scans were recorded in 32 frames (5×2, 4×5, 3×10, 8×30, 5×60, 4×300 und 3×600 s). All attenuation-corrected images were reconstructed to a 128 × 128 × 159 matrix (0.78 × 0.78 × 0.80 mm) using an iterative algorithm (ordered subset expectation maximization 3D; maximum a posteriori; 2 OSEM iterations; 18 MAP iterations, β = 1). Biodistribution studies were obtained from PET data. For evaluation of activity in affected muscles, a volume of interest (VOI) was defined manually. The size was constant and the position of the VOI was first adapted via CT image, so that the VOI did not reach into the tibia or knee joint. Secondly, the VOI was adjusted via PET images around fibula or above the tibia when the leg was strongly swollen. Reproducibility was ensured through two independent analysts (Figure S3). Based on these VOIs, the percentage of injected dose per gram tissue (%ID/g) was calculated [voxel intensity (Bq/g) over the injected dose (Bq) x 100%].

**Metabolite analysis:** Under isoflurane anesthesia, healthy mice were administered [^68^Ga]Ga-DMALTO (11.0 ± 1.6 MBq) through a tail vein and were sacrificed after 5, 10, 15, 20 or 60 min. Blood and urine samples were collected and analyzed via radio-TLC and -HPLC.

**Histology:** After PET/CT scans, mice were perfused with formalin under ketamine/xylazine anesthesia and the *gastrocnemii* were collected, embedded in Tissue-Tek O.C.T Compound and sliced (10 µm) (21). The randomly chosen slices were stained with a BBL Gram Stain Kit (Becton, Dickinson and Company; Franklin Lakes, USA) or, immunostained with antibodies against Ly6G (Biotin anti-mouse Ly-6G, Clone 1A8, BioLegend, CA, USA) or CD68 (Rat anti Mouse CD68:Biotin, Bio-Rad Laboratories, CA, USA).

**Bacterial radiolabeling:** Bacteria were grown overnight shaking at 37 °C in LB. Bacteria were then subcultured into fresh LB and grown until log phase (A_600_ = 0.5) 17, 21. Twenty nmol (10 MBq) [^68^Ga]Ga-DMALTO was added to 1 mL of this bacteria solution (10^8^ CFU) and incubated for 1 h at 37 °C in a shaking incubator. Afterwards, the bacteria were filtered through Microsep Advance Centrifugal Device and washed three times with ice cold PBS. Radioactivity was measured in a 2470 WIZARD^2^ gamma counter from PerkinElmer (Waltham, MA, USA) with a standard dilution (1%) of the injected dose for calculation of uptake. Bacteria were lysed with lysozyme [300 µg/mL lysozyme (Sigma-Aldrich, St. Louis, USA) with 0,5 mM Pefabloc SC (Sigma-Aldrich, St. Louis, USA)] to determine the protein concentration by Bradford protein assay.

**Mammalian cell radiolabeling:** Human monocyte cells THP-1 (ATCC TIB-202) were grown in Gibco RPMI 1640 medium (ThermoFisher Scientific, Waltham, MA, USA) containing 10% fetal calf serum, 50 nM beta-mercaptoethanol and 1% penicillin/streptomycin and 1% L-glutamine, and then seeded onto fibronectin-coated 6 w bell plates (10^6^ cells per well). Cells were differentiated into macrophages with RPMI 1640 medium supplemented with 20% FCS and macrophage colony-stimulating factor (M-CSF, 100 ng/ml) for 4 days 22-24. Subsequently, the differentiation medium was replaced with normal growth media. Cells were incubated with 0.2 nmol (100 kBq/well) [^68^Ga]Ga-DMALTO for 1 h at 37 °C. Following incubation, cells were washed twice with ice cold PBS and lysed in lysis buffer (150 mM NaCl, 1% Triton-X-100, 0.5% sodium deoxycholate, 0.1% sodium dodecyl sulfate, 50 mM Tris, pH 8.0). Cell lysates were counted in a gamma counter, with a standard dilution (1%) of the injected dose for calculation of uptake and the protein concentration was determined by Bradford protein assay.

**GMP-production of [^68^Ga]Ga-DMALTO:** [^68^Ga]Ga-DMALTO was prepared in compliance with GMP for clinical application. The radiosynthesis was performed on a GAIA GMP automated radiosynthesizer (Elysia-raytest, Straubenhardt, Germany) connected to a [^68^Ge]Ge/[^68^Ga]Ga generator (0.74 - 1.85 GBq Galli Ad, IRE-Elit, Fleuru, Belgium) equipped with a sterile, single-use cassette and reagent kit (ABX, Radeberg, Germany). A standardized labeling sequence with 30 nmol of DMALTO was used (for details see Figure S4). The decay corrected radiochemical yield at the end of the synthesis was found to be 75% (59 - 98%) with a coefficient of variation of 0.195. The quality control was performed analogous to the radiochemistry of [^68^Ga]Ga-DMALTO for *in vitro* and small animal PET/CT (≥95% [^68^Ga]Ga-DMALTO). Production batches were further tested for pH (4.0 - 5.5), sterility, bacterial endotoxins (<10 I.E./mL), residual solvents (<50 mg/mL ethanol) and radionuclide purity (γ-spectroscopy, ≥99.999% of Ga-68). For validation of the radio-TLC additional radio-HPLC (see Figure S1 and S2) was performed.

**Clinical study population:** The study group consisted of 8 LVAD recipients (2 women, 6 men; age, 54.9 ± 19.7 years; range 24.9-77.9 years) who had been referred to our institution for an [^18^F]FDG PET/CT. LVAD recipients with end-stage heart failure are particularly prone to device-related infections because the driveline, which connects the external battery pack to the internal pump, provides a pathway for bacterial entry, colonization of device components, and biofilm formation. Symptoms of LVAD infection are nonspecific, while MRI and CT investigations are artifact-prone, and therefore the depth of infection is difficult to evaluate. Hence, LVAD infection has emerged as an accepted clinical indication for [^18^F]FDG scans, where the involvement of internal components of the device indicates elevated risk and justifies surgical revision or even a very risky pump replacement. However, any type of surgery, manipulation for probe sampling, or mechanical tissue irritation by implant components will result in (sterile) activation of the immune system, making the [^18^F]FDG signal nonspecific and difficult to interpret. Hence, this critical patient group was chosen as a very suitable setting for the implementation of bacteria-specific PET imaging with [^68^Ga]Ga-DMALTO, in order to improve upon the [^18^F]FDG signal by providing pathogen-specific imaging. The [^68^Ga]Ga-DMALTO PET/CT scan occurred at an interval of 6.5 ± 12 days [mean interval ± standard deviation (SD); range 1 - 36 days] after the [^18^F]FDG PET/CT. In-house produced, GMP-compatible [^68^Ga]Ga-DMALTO was used clinically according to §13.2b of the German Pharmaceuticals Act, which enables the use of novels radiopharmaceuticals for exploration of specific clinical questions under the physician's responsibility and based on an individual clinical decision. Accordingly, all patients were referred for experimental diagnostics by their treating medical specialists, who faced persistently increased infection parameters despite antibiotic use and an unmet diagnostic challenge that could not be solved sufficiently with standard diagnostic means. Although the possible location of the infectious lesions was frequently known, the intent was to receive more information about the actual bacterial colonization for reasons such as switching antibiotics regime or planning further surgical interventions. Smear results from driveline entry points, systemic inflammatory markers, and duration of antibiotic therapy were recorded for each patient at the time of both PET/CTs. All patients gave written informed consent to receive a [^68^Ga]Ga-DMALTO PET/CT. The clinically-acquired data were then analyzed retrospectively with approval of the local ethics committee of Hannover Medical School (approval no. 9862_BO_K_2021).

**PET/CT imaging acquisition and reconstruction:** All studies were obtained on a dedicated PET/CT system (Siemens Biograph mCT 128 Flow; Siemens Knoxville, TN) equipped with an extended field-of-view PET component, a 128-slice spiral CT component, and a magnetically-driven table optimized for continuous scanning. For [^18^F]FDG PET/CT imaging, 321 ± 16 MBq of [^18^F]FDG were injected intravenously after 6 h of fasting and confirmation that blood glucose levels were less than 8 mmol/L. After an uptake time of 60 min, whole-body PET scans were acquired as previously described 25.

Synthesis and labeling of [^68^Ga]Ga-DMALTO was performed as described above. Following the regulations of the German Pharmaceuticals Act §13.2b, the indication for the exam and labeling of the [^68^Ga]Ga-DMALTO tracers was done under the direct responsibility of the applying physician. [^68^Ga]Ga-DMALTO PET/CT imaging started with a caudocranial low-dose whole-body CT (120kV, mA modulated, pitch 1.2, reconstructed axial slice thickness 5.0 mm), which was performed for attenuation correction of PET acquisitions. Then, PET acquisition was started 10s before intravenous injection of 133 ± 20 MBq [^68^Ga]Ga-DMALTO. The protocol consisted of three consecutive dynamic partial-body scans (head to proximal femora) using continuous bed motion at a speed of 2.0 mm/s for head, 0.6 mm/s for chest and abdomen, and 1.0 mm/s for legs and an additional partial-body scan after an uptake period of 120 min. All studies were reconstructed using time-of-flight and point-spread function TrueX information combined with an iterative algorithm (Ultra HD^®^, Siemens Healthcare; 2 iterations, 21 subsets, matrix 200; zoom 1.0; Gaussian filter of 2.0).

**PET/CT image analysis:** Transaxial PET, CT, and fused PET/CT images were analyzed both visually and semi-quantitatively on a dedicated workstation (syngo.via; V10B; Siemens Healthcare, Erlangen, Germany). PET images were visually evaluated by consensus of two experienced readers (DW and CC) for the presence of focally increased [^18^F]FDG and [^68^Ga]Ga-DMALTO uptake related to LVAD or other infectious foci. A focus was graded as positive if it was significantly elevated versus background in both non-attenuation and attenuation-corrected images to avoid artifacts induced by dense implant material. For localization of LVAD-specific infection, the LVAD was subdivided into 4 components according to the International Society for Heart and Lung Transplantation recommendations 26: (1) driveline entry point, (2) subcutaneous driveline pathway, (3) pump pocket, and (4) outflow tract.

Lesion tracer uptake was quantified by mean standardized uptake value (SUVmean) and SUVpeak 15, 30, 45, and 120 min after injection of [^68^Ga]Ga-DMALTO and 60 min after injection of [^18^F]FDG, respectively. For calculation of the SUV an individual 3D VOI (typically 10 mm in diameter) was manually placed around a focus on co-registered transaxial PET/CT images. For calculation of lesion target-to-background ratio (TBR), the SUVpeak of each lesion was divided by the SUVmean of blood-pool for TBR_blood-pool_ and by the SUVmean of gluteal muscles for TBR_muscle_. The nonspecific background in blood-pool was measured as mean from 3 regions of interest of fixed size (10 mm) placed in the mid lumen of the superior vena cava. Additionally, SUVs were obtained from abdominal wall contralateral to driveline entry point, liver, spleen, bone marrow, gluteal muscles, kidneys, and bladder.

**Statistics:** Statistical evaluation was performed with GraphPad Prism (version 5.0, La Jolla CA, USA) and SciPy Version 1.7.3 27. For the *in vitro* and *in vivo* data a two-tailed Student's unpaired *t*-test was chosen. Variances strongly deviated between two groups; therefore, a Welch's correction was done. For patient data continuous variables are expressed as mean ± SD. Categoric variables are presented with absolute and relative frequencies. Normal distribution was checked by a Shapiro-Wilk test. For between-group comparisons of parametric continuous data, *P* values were calculated from a paired, two-sided Student`s *t*-test. The Spearman's rank correlation coefficient *rho* was used to assess the relationship between PET findings and clinical data (e.g. leukocyte number, C-reactive protein, and duration of antibiotic therapy). Level of significance was determined for all tests to p < 0.05 with *** p ≤ 0.001, ** p ≤ 0.01, * p ≤ 0.05, and ns p ≥ 0.05.

## Results

### The positron-emitting, bacteria-specific radiotracer [^68^Ga]Ga-DMALTO shows suitable characteristics for *in vivo* PET imaging

DOTA-maltohexaose was coupled to the bifunctional chelator DOTA (Figure S5). Gallium-68 labeling was performed using purified eluate of a [^68^Ge]Ge/[^68^Ga]Ga generator in 1.5M HEPES buffer at 100 °C for 10 min. Kinetics were fast and radiochemical yield was high (96.9 ± 3.3%), obviating the need for further purification before *in vitro* and *in vivo* animal applications. The apparent molar radioactivity of [^68^Ga]Ga-DMALTO was 10 - 20 GBq/µmol at the end of synthesis.

**Figure 1:**
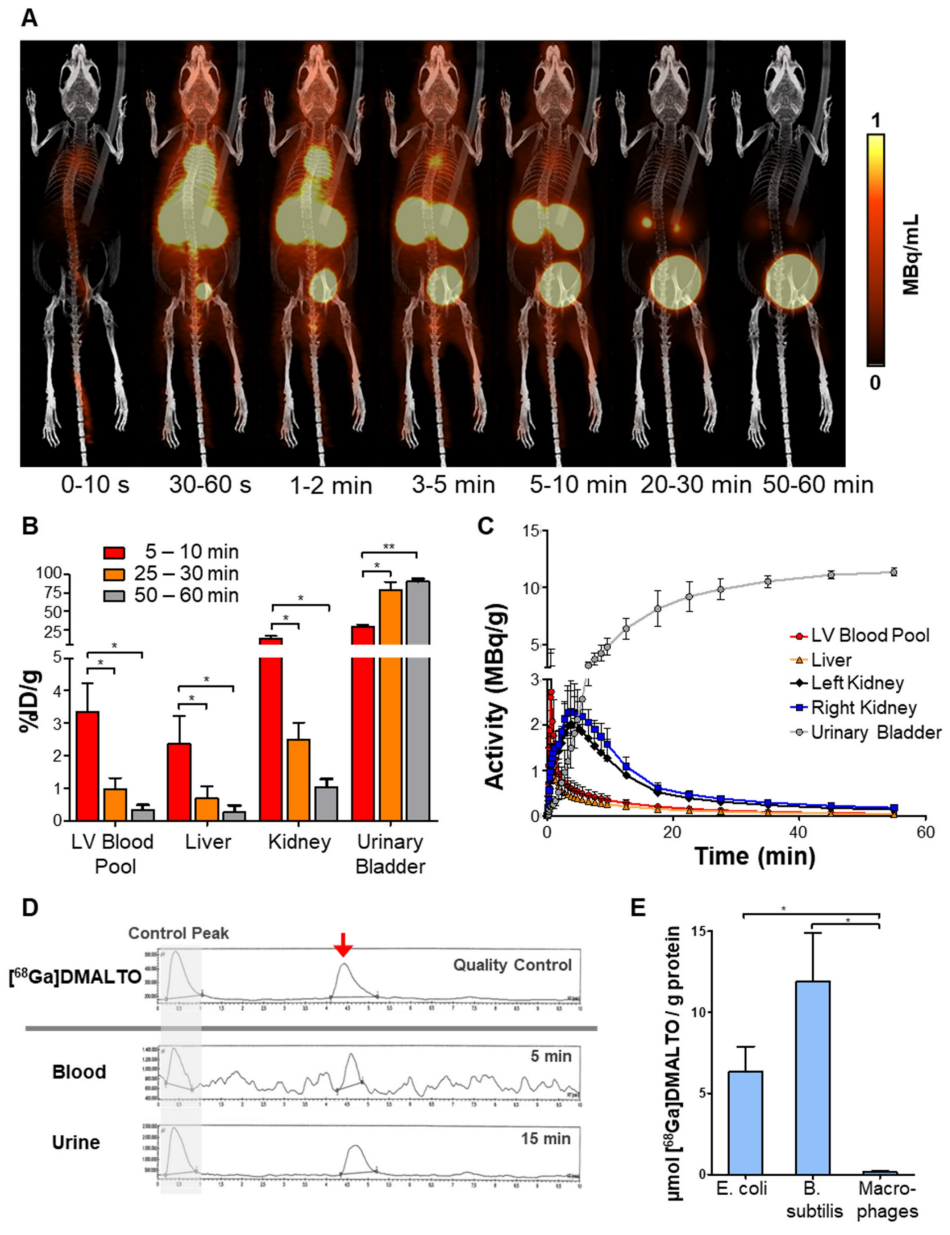
***In vivo* characterization of [^68^Ga]Ga-DMALTO.** (**A**) Representative 3D lateral maximum intensity projection fused PET/CT images at different time intervals after [^68^Ga]Ga-DMALTO injection. (**B**) Tracer uptake (%ID/g) in blood and excreting organs at the respective time intervals. (**C**) Detailed time-activity curves of selected organs. (**D**) Representative high-performance liquid chromatograms from quality control of synthesized [^68^Ga]Ga-DMALTO (top), blood (5 min), and urine (15 min) after *in vivo* injection (bottom) with matching peaks for the tracer product (red arrow) that are comparable to the early control peak (before retention on the HPLC column, indicated with grey background), and the absence of metabolite peaks. (**E**) *In vitro* tracer uptake in *E. coli* and *B. subtilis* compared to human macrophages. (*p ≤ 0.05).

*In vivo* kinetics were determined by PET/CT in healthy mice. Following administration, dynamic images displayed rapid renal excretion of [^68^Ga]Ga-DMALTO and fast clearance of nonspecific background activity (Figure 1A). Accumulation in the kidneys peaked at 4 min and in the absence of bacterial infection, most tracer was localized to the urinary bladder, with >75 %ID/g (percentage of total injected dose per gram tissue) there at 30 min and >90 %ID/g there after 60 min. No organ showed physiologic accumulation of the radiotracer and blood activity remained very low (Figure 1B - C, n = 3). In addition, no radiolabeled metabolites were detected in blood or urine according to radio-TLC and -HPLC analysis (Figure 1D). Rapid renal clearance, the absence of nonspecific accumulation, and radiotracer stability support feasibility of [^68^Ga]Ga-DMALTO as an *in vivo* tracer of bacterial infections. By contrast, *in vitro* radiotracer uptake assays demonstrated selective accumulation of [^68^Ga]Ga-DMALTO in Gram-negative (*E. coli*) and Gram-positive (*B. subtilis*) bacteria. This uptake was not observed in cultured human macrophages, supporting selectivity bacteria (Figure 1E).

### [^68^Ga]Ga-DMALTO identifies bacterial infection in mice

**Figure 2:**
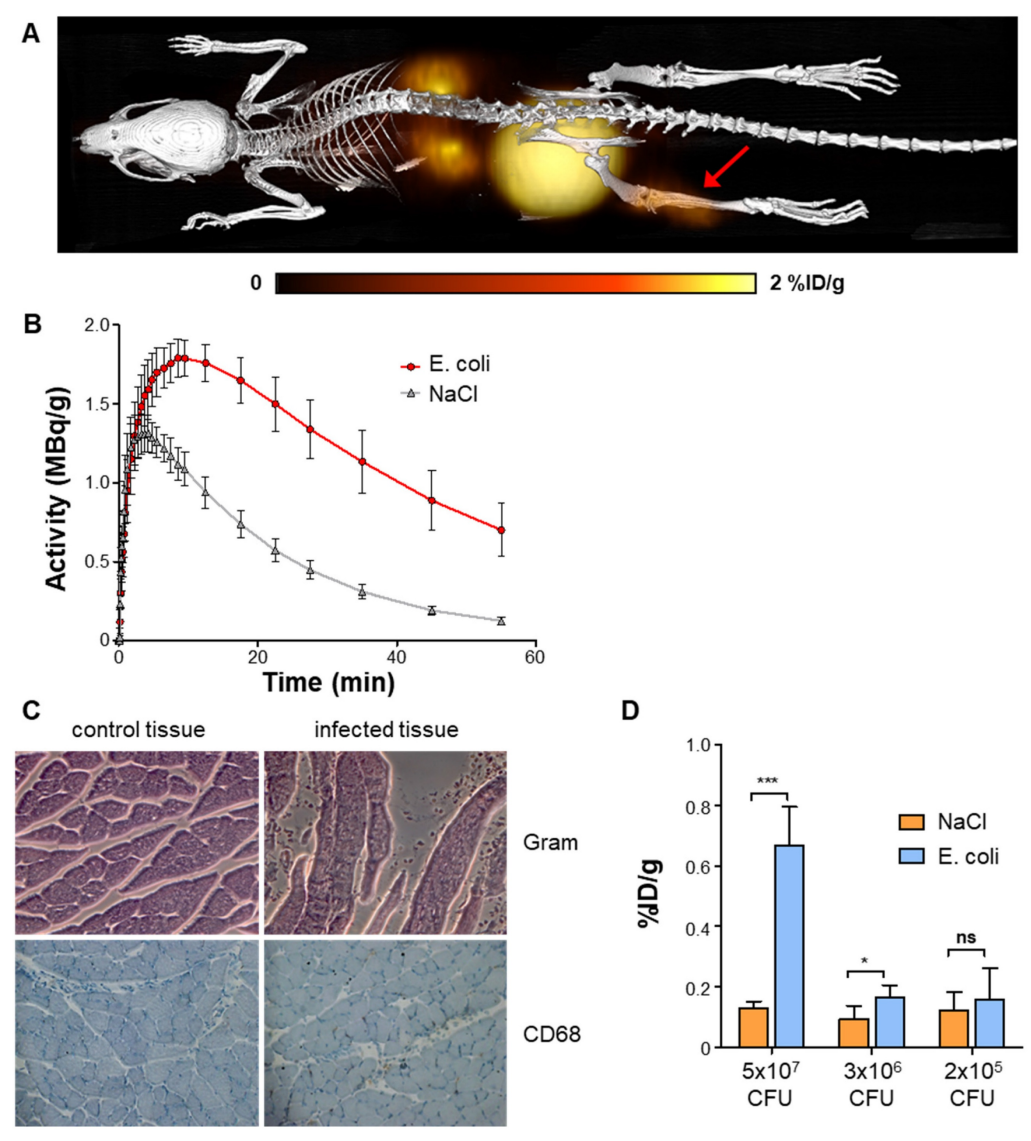
***In vivo* PET using [^68^Ga]Ga-DMALTO in a mouse model of hindlimb bacterial infections.** (**A**) Representative 3D dorsal maximum intensity projection fused PET/CT image at 60 min after [^68^Ga]Ga-DMALTO injection, showing uptake in the *E. coli* infected hindlimb (red arrow). (**B**) Time-activity-curves in %ID/g for infected (*E. coli*) and negative control (NaCl) *gastrocnemius* muscles. (**C**) Histopathology of infected (right) and control (left) muscle, stained for bacteria (Gram, top) and monocytes (CD68, bottom)*.* (**D**) [^68^Ga]Ga-DMALTO accumulation in %ID/g using decreasing amounts of *E. coli* CFU.

*In vivo* PET/CT was performed 24 h after *E. coli* inoculation into the *gastrocnemius* muscle of mice, at a time of clinically apparent unilateral hindlimb infection (Figure 2A). Focally elevated uptake of [^68^Ga]Ga-DMALTO was observed at the site of infection compared to the saline-injected contralateral hindlimb as early as 2 min after injection, remaining significantly elevated until the end of dynamic imaging (Figure 2B; 0.70 ± 0.15 *vs.* 0.13 ± 0.02 %ID/g at 60 min, p < 0.0001, n = 10). Histopathology confirmed the presence of bacteria, tissue injury, and monocyte infiltration at the site of infection (Figure 2C). The *in vivo* [^68^Ga]Ga-DMALTO signal was dependent on the amount of bacteria in the target region (Figure 2D), as signal decreased with decreasing doses of inoculated *E. coli* CFU and remained detectable down to a concentration of 3x10^6^ CFU, when compared to the non-infected hindlimb (p = 0.0143, n = 6).

**Figure 3:**
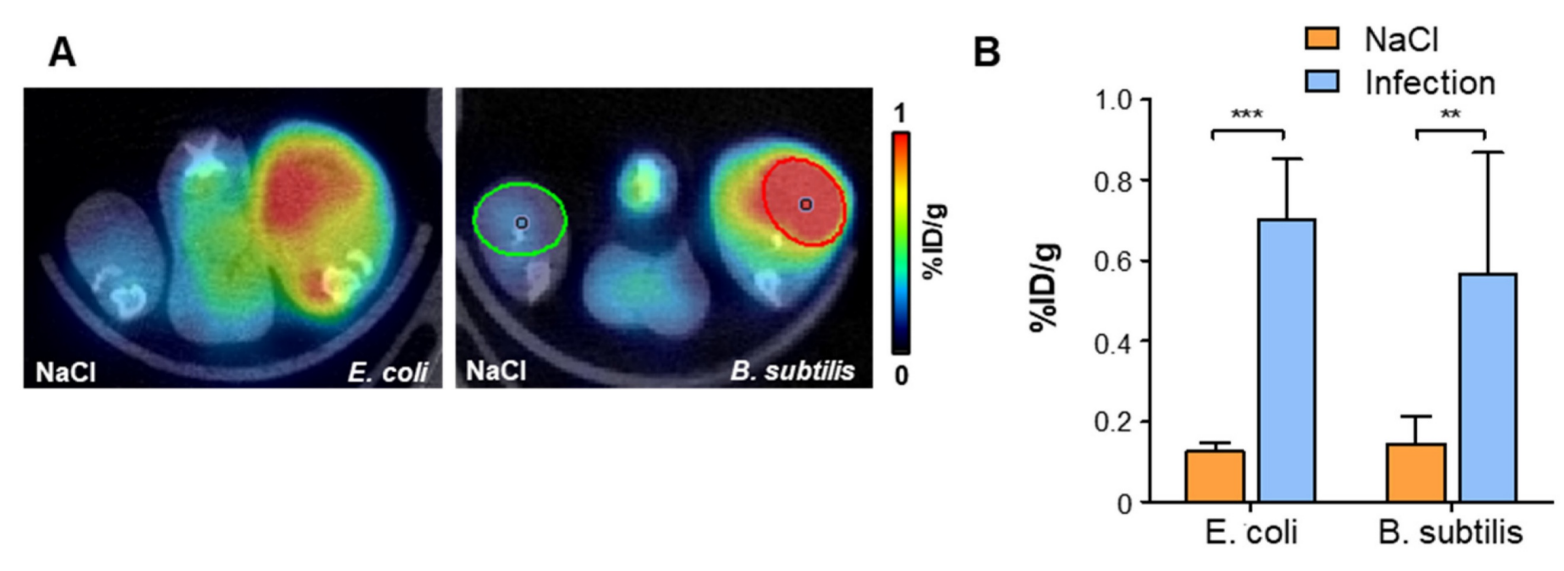
***In vivo* PET using [^68^Ga]Ga-DMALTO in comparison with Gram-positive bacterial infections** (**A**) Representative transaxial PET/CT images showing significant unilateral hindlimb [^68^Ga]Ga-DMALTO accumulation at sites of *E. coli* and *B. subtilis* infection. (**B**) [^68^Ga]Ga-DMALTO accumulation in %ID/g in infected *(E. coli* or *B. subtilis*) and contralateral (NaCl) hindlimbs. (***p ≤ 0.001, **p ≤ 0.01, *p ≤ 0.05, ns = p ≥ 0.05).

To determine the feasibility of using [^68^Ga]Ga-DMALTO for detection of Gram-positive bacteria, a second series of animals was studied using *B. subtilis* (Figure 3A - B). Similar to *E. coli*, significantly elevated uptake of [^68^Ga]Ga-DMALTO was observed in infected muscle (0.57 ± 0.30 *vs.* 0.14 ± 0.07 %ID/g, p = 0.0019, n = 10).

### Unlike the glucose analogue [^18^F]FDG, [^68^Ga]Ga-DMALTO is specific for bacterial infection versus aseptic hindlimb inflammation

**Figure 4:**
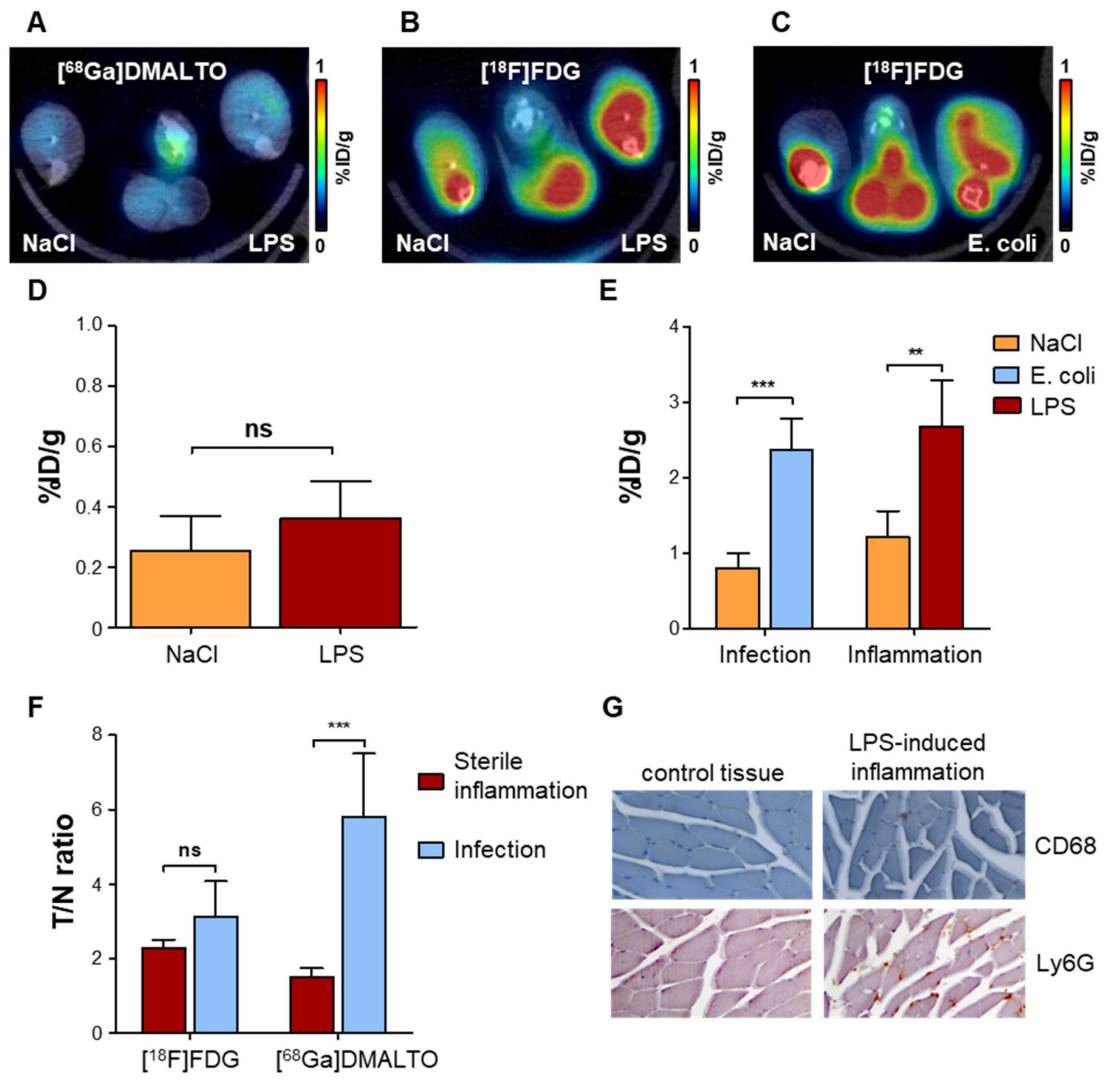
***In vivo* PET using [^68^Ga]Ga-DMALTO for specific detection of bacterial infection versus sterile inflammation.** (**A**) Representative transaxial PET/CT image showing the absence of unilateral [^68^Ga]Ga-DMALTO signal elevation in LPS-induced aseptic inflammation. (**B**) Representative transaxial PET/CT image showing elevated [^18^F]FDG uptake in aseptically inflamed hindlimb muscle, along with non-specific uptake in bone marrow and remote muscles. (**C**) Representative transaxial PET/CT image showing elevated [^18^F]FDG uptake in *E. coli*-infected hindlimb muscle, along with non-specific uptake in bone marrow and remote muscles, similar to aseptic inflammation of panel C. (**D**) [^68^Ga]Ga-DMALTO uptake in %ID/g for aseptically inflamed versus contralateral hindlimb. (**E**) [^18^F]FDG uptake in %ID/g for models of aseptic inflammation and *E. coli*-induced infection. (**F**) Relative uptake differences (target/nontarget ratio, T/N ratio) for [^18^F]FDG and [^68^Ga]Ga-DMALTO in both models. (**G**) Histopathology of aseptically inflamed (right) and control (left) muscle, stained for monocytes (CD68, top) and granulocytes (Ly6-G, bottom). (***p ≤ 0.001, **p ≤ 0.01, *p ≤ 0.05, ns = p ≥ 0.05).

To determine whether [^68^Ga]Ga-DMALTO could distinguish between a bacterial infection and aseptic inflammation, we injected lipopolysaccharide (LPS) intramuscularly into the *gastrocnemius* muscle of mice to generate a local inflammatory response. In this model, serial [^18^F]FDG imaging revealed sustained inflammation over 1-7 d after LPS administration, with maximal PET signal at day 3 (Figure S6). We compared uptake of [^68^Ga]Ga-DMALTO with uptake of [^18^F]FDG. [^68^Ga]Ga-DMALTO showed low uptake at the site of LPS (Figure 4A), which was not statistically different from the non-inflamed contralateral site (Figure 4D; 0.36 ± 0.12 *vs.* 0.25 ± 0.12 %ID/g, p = 0.1844, n = 5). By contrast, [^18^F]FDG accumulation was significantly elevated in both aseptic inflamed hindlimbs (Figure 4C) and upon *E. coli* infection (Figure 4D). In fact, the amplitude of [^18^F]FDG signal was not different between aseptic inflammation (2.68 ± 0.60 *vs.* 1.21 ± 0.35 %ID/g, p = 0.0015, n = 5) and bacterial infection (2.38 ± 0.41 *vs.* 0.81 ± 0.19 %ID/g, p < 0.0001, n = 5; Figure 4D). Of note, nonspecific accumulation of [^18^F]FDG in muscle and bone marrow further complicated the isolation of the inflammation signal, while [^68^Ga]Ga-DMALTO images generally showed very low background. Unlike [^18^F]FDG**,** [^68^Ga]Ga-DMALTO uptake was significantly different between bacterial infection and aseptic inflammation (Figure 3F; p ≤ 0.0001). Immunostaining confirmed the presence of CD68-positive macrophages and Ly6G-positive granulocytes in the LPS-injected muscle (Figure 4G).

### GMP-compatible radiosynthesis of [^68^Ga]Ga-DMALTO is feasible

For compliance with GMP, further optimization of the radiosynthesis was needed to provide [^68^Ga]Ga-DMALTO for clinical application in patients. With an automated radiosynthesizer (GAIA^©^, Elysia-Raytest, Germany), a validated, robust, and reproducible method was established (RCY of 75% with a coefficient of variation of 0.195). Based on the monographs of the European Pharmacopeia (Ph. Eur.), a complete quality control was developed and validated. Each production batch was tested for radiochemical identity and purity (17.0 ± 4.3 MBq/mL, 98.0 ± 1.6% of [^68^Ga]Ga-DMALTO), radionuclide purity by γ-spectroscopy (≥99.999% of [^68^Ga]Ga), pH (4.9 ± 0.5), sterility, bacterial endotoxins (<1 I.E./mL), and residual solvents (39.1 ± 8.1 mg/mL ethanol). All results were within the required specifications, enabling clinical application of the radiotracer.

### Biodistribution of [^68^Ga]Ga-DMALTO in humans confirms renal clearance and low background tissue signal

**Figure 5:**
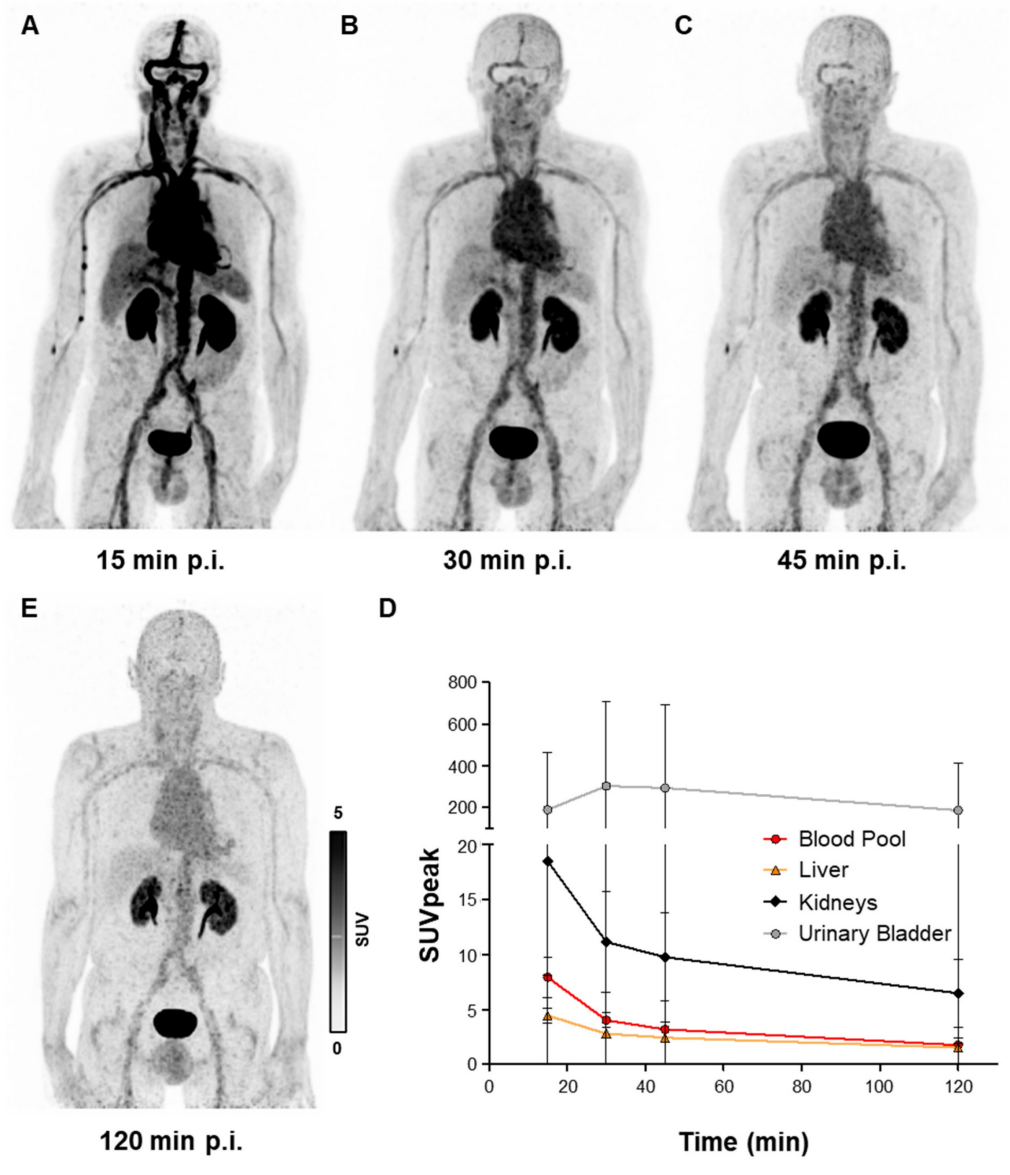
** Human biodistribution of [^68^Ga]Ga-DMALTO.** Anterior maximum intensity projection PET images of a 62-year-old male LVAD recipient, showing biodistribution of [^68^Ga]Ga-DMALTO (**A**) at 15 min, (**B**) 30 min, (**C**) 45 min, and (**D**) 120 min post injection (p.i.). (**E**) Time-activity curves of blood and excreting organs.

Early clinical application of [^68^Ga]Ga-DMALTO was in patients with LVAD infection who showed persistently elevated infection parameters despite antibiotic use. All studied LVAD recipients (n = 8) tolerated [^68^Ga]Ga-DMALTO examination well. No drug-related pharmacologic effects or physiologic responses occurred and no patient reported any new symptoms during injection and 2.5 h of follow-up. In accordance with biodistribution of [^68^Ga]Ga-DMALTO in mice, dynamic [^68^Ga]Ga-DMALTO PET images in patients showed renal clearance of the tracer with a peak activity in urinary bladder after 30 min. Blood-pool clearance was markedly slower in humans versus mice, emphasizing the need for later imaging time points to allow for sufficient reduction of background signal. Due to lack of specific uptake in human tissue, low signal was present outside of vascular structures and excreting organs from 30 min after [^68^Ga]Ga-DMALTO injection (Figure 5A - E).

### [^68^Ga]Ga-DMALTO identifies bacterial LVAD infection

**Figure 6:**
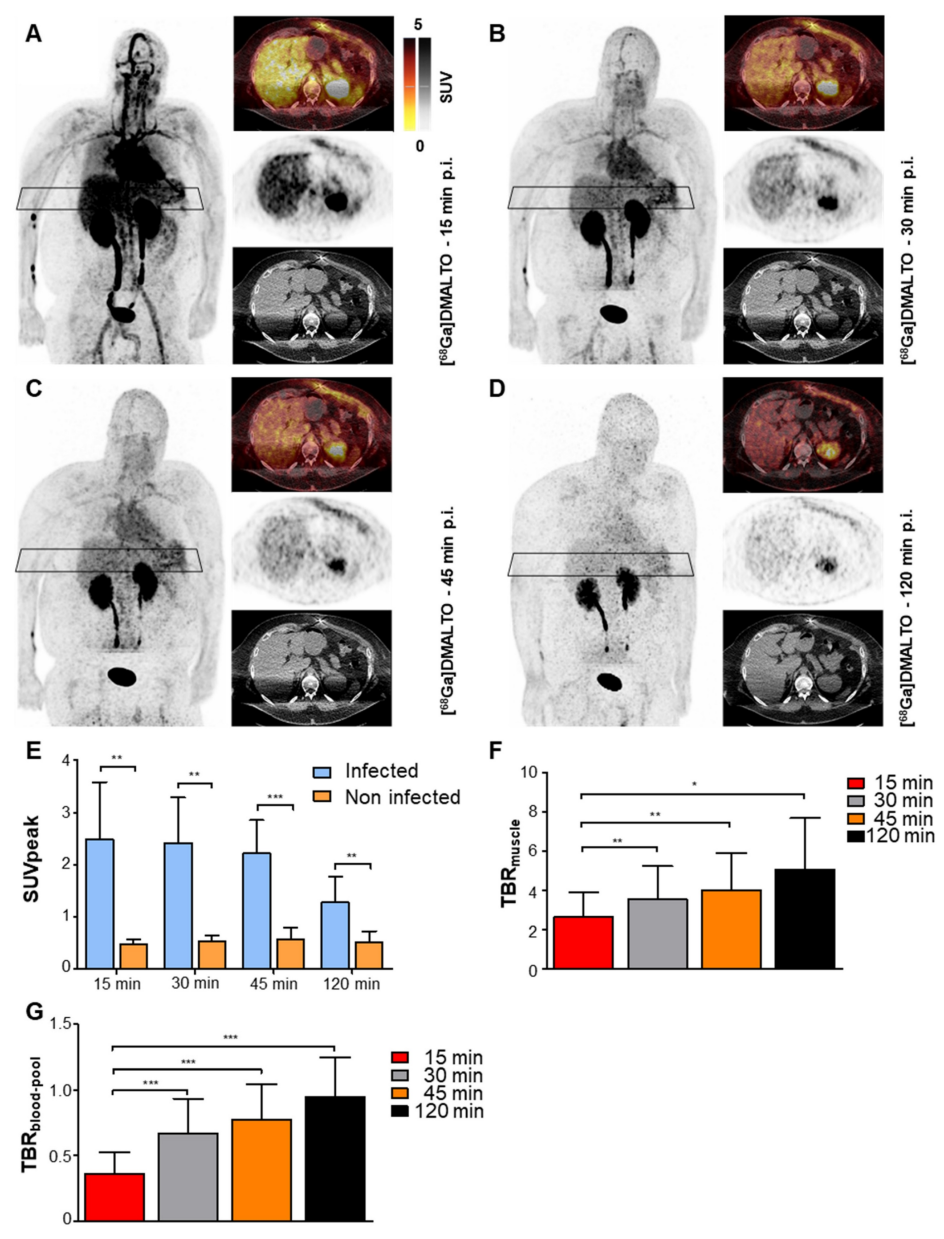
** [^68^Ga]Ga-DMALTO uptake in bacterial LVAD infection.** (**A-D**) Anterior maximum intensity projections along with transaxial PET, CT, and fused PET/CT images of a male LVAD recipient with *Pseudomonas aeruginosa* and *Proteus mirabilis* device infection along the subcutaneous driveline pathway, at 15 min (**A**), 30 min (**B**), 45 min (**C**), and 120 min (**D**) p.i. (**E**) Comparison of [^68^Ga]Ga-DMALTO uptake values at driveline entry point (infected) and at the contralateral abdominal wall (non-infected) in all patients with a microbiologically proven bacterial infection at the driveline entry point. (**F**) TBR_muscle_ and (**G**) TBR_blood-pool_ of [^68^Ga]Ga-DMALTO at the infected driveline entry point.

Seven of eight LVAD recipients had antibiotic therapy which started prior to [^68^Ga]Ga-DMALTO PET/CT (average 10 days; range, 1 - 35 days). Pre-test probability for driveline infection was high in the majority of patients (n = 7) based on the following criteria: 1) proof of bacteria at driveline entry point smears, 2) presence of local signs of infection e.g. redness, swelling, local hyperthermia and exudation, 3) detectable [^18^F]FDG-uptake at driveline entry point at a prior PET scan. One LVAD recipient met none of the criteria and served as a low probability comparison. All patients with high pretest probability demonstrated visually detectable, focal [^68^Ga]Ga-DMALTO uptake at the driveline entry point, suggestive of the presence of bacteria and LVAD-specific infection (Figure 6A - D).

A total of 11 different bacterial strains were identified in driveline entry point smears. Four patients suffered from mixed infections with 2 or more pathogens, with *Staphylococcus* spp. being the most frequent (45%) and demonstrating - in case of *S. aureus* - the highest SUV values at driveline entry points. Corresponding to renal tracer clearance, the absolute intensity of focal [^68^Ga]Ga-DMALTO uptake at driveline entry points decreased over time (Figure 6E), while contrast to background (TBR_blood-pool_ and TBR_muscle_) increased (Figure 6F - G).

### Bacteria-specific [^68^Ga]Ga-DMALTO signal is consistent with host immune response driven [^18^F]FDG signal in bacterial LVAD infection, but less intense

**Figure 7:**
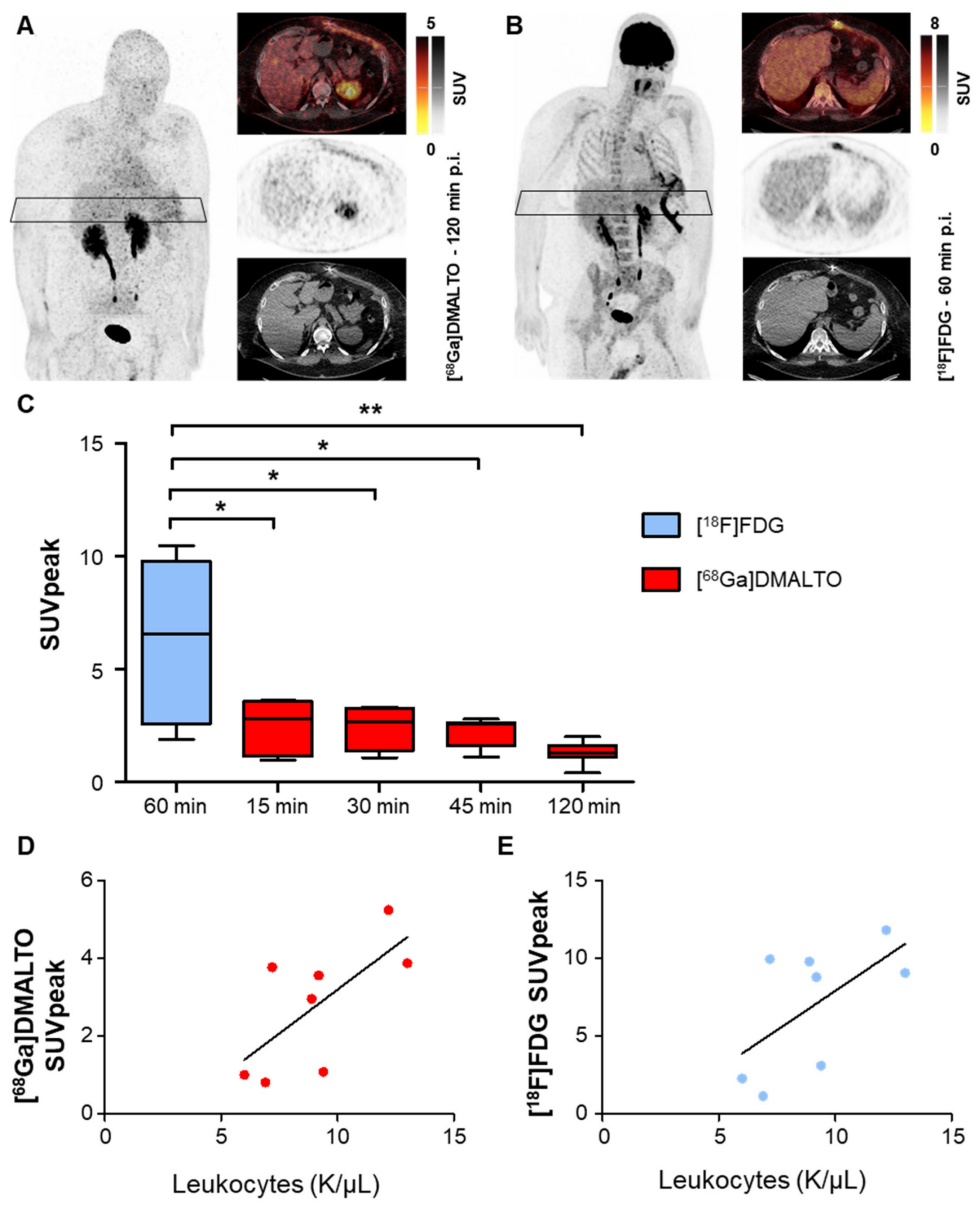
**[^68^Ga]Ga-DMALTO- and [^18^F]FDG-PET/CT in LVAD infection.** Maximum intensity projections, PET, CT, and fused PET/CT images of (**A**) [^68^Ga]Ga-DMALTO at 120 min and (**B**) [^18^F]FDG at 60 min p.i. in the same individual with LVAD infection shown in Figure 5, for comparison. (**C**) Tracer uptake of [^68^Ga]Ga-DMALTO and [^18^F]FDG at the driveline entry point at different time points *post injection* in patients with proven bacterial driveline entry point infection. (**D**) Correlative analysis of leukocyte counts and [^68^Ga]Ga-DMALTO (**E**) as well as [^18^F]FDG uptake at subcutaneous driveline pathway in patients with infection of this LVAD component.

Visually detectable focal [^68^Ga]Ga-DMALTO uptake at the site of infected LVAD driveline co-localized with elevated [^18^F]FDG signal in all patients, as seen in a representative example in Figure 7A - B. Focal tracer uptake of [^18^F]FDG was significantly higher in infected LVAD components than the bacteria-specific [^68^Ga]Ga-DMALTO signal at all time points (Figure 7C). Of note, correlative analysis revealed that [^68^Ga]Ga-DMALTO uptake along the subcutaneous driveline pathway correlated with systemic inflammatory markers at all timepoints (e.g., [^68^Ga]Ga-DMALTO uptake 15 min post injection, C-reactive protein, *rho* = 0.71, p = 0.047; leucocyte count, *rho* = 0.74, p = 0.037, *vs.* [^18^F]FDG uptake 60 min. post injection, C-reactive protein, *rho* = 0.69, p = 0.058; leucocyte count, *rho* = 0.50, p = 0.207; Figure 7D - E).

## Discussion

The presented work demonstrates the feasibility of a PET-radiotracer, [^68^Ga]Ga-DMALTO, for specific and selective identification of bacteria in a translational setting from bench through experimental animals towards early human application. Robust synthesis of the radiopharmaceutical was established and adjusted to GMP requirements. *In vivo* imaging enabled the detection of bacterial infections in a mouse model and in LVAD patients as a typical clinical setting for noninvasive molecular imaging. The signal from the novel bacterial-specific radiotracer enabled discrimination between bacterial infection and aseptic inflammation.

Such specific *in vivo* detection of the localization and density of bacterial pathogens holds the potential to refine and individualize the management of infectious disease. Concurrent, clinically-employed radiopharmaceuticals for molecular imaging of infection and inflammation, such as radiolabeled white blood cells or the glucose analogue [^18^F]FDG, are mostly focused on immune cells, which define the host response to pathogens 3. While this approach provide a robust signal that supports clinical feasibility for lesion detection, targeting the host response rather than the pathogen itself may limit diagnostic accuracy 28. Aseptic inflammation or reparatory immune responses are difficult to distinguish from pathogen-induced immune activation, limiting specificity 29, 30. By imaging the host response to infection, sensitivity may also be limited in immunosuppressed patients or other immunomodulatory conditions. Moreover, accumulation in physiologic niches of immune cells, such as the bone marrow, spleen, or lymph nodes may complicate image interpretation 29-31. The standard PET radiopharmaceutical for clinical workup of certain infectious and inflammatory conditions is [^18^F]FDG, which is a glucose analog that enters mammalian cells via glucose transporters (GLUT). It is then phosphorylated by hexokinase, but not further metabolized, leading to metabolic trapping. Due to their increased glycolysis, inflammatory cells take up [^18^F]FDG avidly 29, 32, 33. By contrast, maltohexaose, a maltodextrin, is used only by bacteria for energy metabolism, where it is taken up via the maltodextrin transporter pathway. This transport system is absent in mammalian cells, turning maltohexaose derivatives, such as [^68^Ga]Ga-DMALTO, into highly specific markers for bacterial pathogens 34. However, due to prior experimental work, some specifics of maltohexaose handling need to be considered even in bacteria. While the maltodextrin transporter belongs to the family of selective ATP-binding-cassette (ABC)-transporters, it provides transmembraneous passage only to non-modified maltodextrins 15, 35, 36. Due to radiosynthesis of [^68^Ga]Ga-DMALTO, maltohexaose is altered at the reducing end, thereby limiting membrane transport. Yet, maltose-binding protein (MBP) is a highly abundant protein and is available to bind numerous variants of maltodextrins. Thereby, [^68^Ga]Ga-DMALTO does not pass the cytoplasmic membrane for intracellular accumulation but remains bound to MBP on the surface. Nevertheless, as shown in our work, this is sufficient for specific visualization via PET/CT. Of note, serum stability, a potential concern for other maltodextrin-based radiopharmaceuticals 15, was not an issue for [^68^Ga]Ga-DMALTO as shown by lack of metabolites in our study.

In prior experimental imaging work in preclinical models, maltohexaose was radiolabeled using fluorine-18, technetium-99m, or fluorescence markers 13, 14, 17. While this enabled specific detection of infectious foci in rodents, with a target signal strength that was comparable to that of the present study, non-specific background signal was higher than in our current [^68^Ga]Ga-DMALTO work, and a direct comparison of aseptic inflammation and bacterial infection was not performed. Accordingly, limited detection sensitivity and specificity are a possible explanation why the previously tested maltohexaose tracers have not yet been successfully translated to humans. Our data, on the other hand, show that labeling of [^68^Ga]Ga-DMALTO with the radiometal [^68^Ga]Gallium provides a tracer that is rapidly cleared via the kidneys in mice, enabling imaging with low background. Additionally, [^68^Ga]Ga-DMALTO shows specific uptake in Gram-positive and Gram-negative bacteria, as shown by us in *E. coli* and *B. subtilis*, and by others in other clinically-relevant pathogens such as *Pseudomonas aeruginosa* or *Staphylococcus aureus* both as planktonic bacteria and within biofilms 13, 15, 17. These data support the notion that [^68^Ga]Ga-DMALTO may serve as a broad range marker of infection not only in experimental animals but also in humans.

For clinical testing, we chose LVAD infection as an accepted indication for PET imaging. Here, the driveline, which connects the external controller with the internal pump, provides an entry point and pathway for the spread of bacteria, leading to infections that are difficult to manage. Infectious spread to internal device components is associated with poor outcome 2, 37. The driveline exit site is accessible and can be swabbed for microbiological testing. This offers the opportunity for image signal validation - as demonstrated in our pilot group - in which all drivelines infected by various species were clearly visualized.

Our first-in-human application of [^68^Ga]Ga-DMALTO provided other valuable insights. First, the blood-pool clearance in our patients was slower compared to mice, requiring adjustment of the optimal imaging time point. Whether this observation reflects species-specific differences or effects of advanced heart failure on renal clearance remains to be determined and should be evaluated in future studies including healthy subjects and other patient cohorts. Second, signal strength for [^68^Ga]Ga-DMALTO was lower when compared to [^18^F]FDG in patients. This may reflect the density of immune cells at the site of infection taking up [^18^F]FDG which is presumably larger than that of only the infectious agent taking up [^68^Ga]Ga-DMALTO.

It should be noted that the LVAD patients had a longstanding history of slowly evolving, chronic infections with multiple prior therapies, including antibiotic therapy at the time of imaging. All of this may have influenced the bacteria-specific [^68^Ga]Ga-DMALTO signal by reducing the number of viable pathogens, while the host immune response identified by the [^18^F]FDG signal was still active. In our small group of patients, results influenced clinical decisions on an individual level, where detected foci of [^68^Ga]Ga-DMALTO accumulation were targeted by surgical revision if possible, or led to prolonged antibiotic therapy.

The initial human studies provide a foundation and generate further hypotheses for follow-up prospective clinical work, which will need to explore [^68^Ga]Ga-DMALTO signal strength in humans in more depth, including e.g. healthy subjects, settings of untreated focal infection, and serial imaging prior to, and in response to, anti-infectious therapy. Such work will then help to define the potential future clinical role of direct bacteria-targeted imaging. With reference to the lower signal strength for [^68^Ga]Ga-DMALTO compared to [^18^F]FDG in our patients, it should be noted that a weaker signal may limit the sensitivity for detection in this setting, but at the same time, it may be specific for the presence of bacteria, and thereby be differently linked to outcome and to the benefit of certain therapeutic interventions. Ultimately, and given the advent of ultra-sensitive, very low dose total-body PET systems 38, a combination of pathogen-targeted and host immune system-targeted tracers may be employed for an in-depth analysis of the host-pathogen interface. This approach may provide even more personalized, non-invasive monitoring of anti-infective therapy and guidance of individualized therapy, providing a faster and more direct readout of treatment efficacy than conventional diagnostics such as biopsy and cell culture. While the half-life of [^68^Ga]Gallium limits late time point imaging, [^68^Ga]Gallium labeled radiotracers typically benefit from rapid renal clearance and low background signal due to low lipophilicity of the radiometal complexes. Furthermore, the DOTA-chelator of [^68^Ga]Ga-DMALTO enables novel therapy approaches with β-emitting radionuclides (like yttrium-90 and lutetium-177) or α-emitting radionuclides (like actinium-225 and bismuth-213) are potential future theranostic applications in patients similar to those in oncology for complex infections where standard therapies do not work anymore. Radioimmunotherapy has, for example, been shown to be effective against infections and biofilms *in vitro* and in *in vivo* mouse models 39-41, which might be transferable to other radiotracers and enable bacteria-specific radiotheranostics.

In conclusion, our translational work establishes an easy-to-synthesize radiolabeled maltodextrin, [^68^Ga]Ga-DMALTO as a clinically-feasible and valid radiopharmaceutical for specific, targeted PET imaging of bacterial pathogens in LVAD patients. This lays the groundwork for subsequent efforts to determine the clinical benefit of bacteria-targeted *in vivo* imaging in individualized infection medicine.

## Supplementary Material

Supplementary figures.

## Data Availability

Data are available in the main text or supplementary materials. Additional source data will be provided by the corresponding author upon individual request.

## Abbreviations

[^18^F]FDG: 2-^18^Fluoro-2-deoxy-D-glucose
[^68^Ga]Ga-DMALTO: [^68^Ga]Gallium-DOTA-maltohexaose
ABC: ATP-binding-cassette
CFU: colony forming units
CT: computed tomography
GLUT: glucose transporter
GMP: good manufacturing practice
HPLC: high performance liquid chromatography
ID: injected dose
LPS: lipopolysaccharide
LVAD: left ventricular assist device
MBP: maltose-binding protein
MRI: magnetic resonance imaging
PET: positron emission tomography
Ph. Eur.: European Pharmacopeia
p.i.: post injection
RCY: radiochemical yield
SD: standard deviation
SPECT: single photon emission computed tomography
SUV: standardized uptake value
TBR: target-to-background
TLC: thin layer chromatography
T/N: target/nontarget
VOI: volume of interest
