# Supplementary material for: Specific imaging of bacterial infection: a translational approach using positron emission tomography and gallium-68-labeled maltohexaose
Source: Theranostics. 2026 Mar 30;16(11):5788–803. doi: 10.7150/thno.125904 (PMC13141696; doi:10.7150/thno.125904)
Supplement: Supplementary file 1 — Supplementary figures. [file thnov16p5788s1.pdf]

Title: Specific imaging of bacterial infection:

A translational approach using positron emission tomography

and gallium-68-labeled maltohexaose

Authors:

Sophie M. Wegener[1*], Desiree Weiberg[1], James T. Thackeray[1], Christoph P. Czerner[1], Jasmin S. Hanke[2], Jan D. Schmitto[2], Geerd-Jürgen Meyer[1], Jens P. Bankstahl[1], Arjang Ruhparwar[2], Frank M. Bengel[1] and Tobias L. Ross[1]

SUPPLEMENTARY FIGURES

Validation of the analytical method for quality control of [^{68}Ga]Ga-DMALTO via radio-HPLC

Radio-TLC was validated with radio-HPLC [NUCLEODUR NH2-RP HPLC column from Macherey-Nagel (Düren, Germany)] with a gradient of 0 – 10 min of 50 – 100% citrate buffer and 50 – 0% acetonitrile and 10 – 13 min 100% citrate buffer. The retention time of [^{68}Ga]Ga-DMALTO is 8.1 min and the retention time of free [^{68}Ga]Gallium is 1.9 min. The control peak at a retention time of 0.1 min is a reference peak, which comes from a first activity detector pass of the full sample without any retention on the HPLC column. The subsequent time course then reflects radioactivity after retention on the HPLC column. Due to retention and

dilution, the following peaks are not exactly identical to the early control peak, but the timing, height, and width convincingly show that no [^{68}Ga]Gallium-complexes remained on the HPLC column.

A

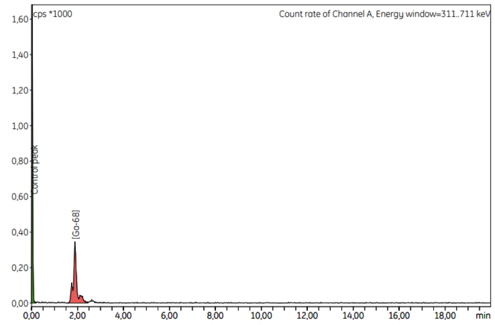

Integration Count rate of Channel A, Energy window=311..711 keV

Substanz	R/T min	Typ	Fläche Counts	%Fläche %
Control peak	0,08	DD(M)	4267,000	55,21
[Ga-68]	1,88	DD(M)	3461,000	44,79

B

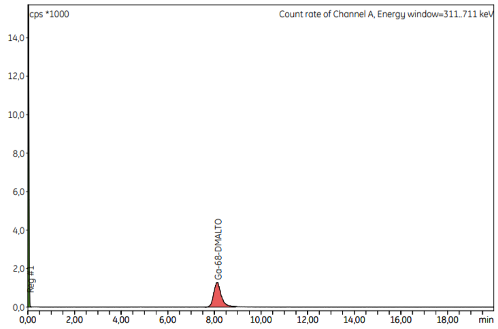

Integration Count rate of Channel A, Energy window=311..711 keV

Substanz	R/T min	Typ	Fläche Counts	%Fläche %
Reg #1	0,07	DD(M)	37036,00	56,22
Ga-68-DMALTO	8,12	DD(M)	28842,00	43,78

C

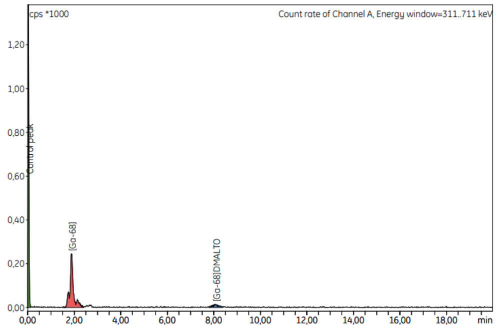

Integration Count rate of Channel A, Energy window=311..711 keV

Substanz	R/T min	Typ	Fläche Counts	%Fläche %
Control peak	0,05	DD(M)	3439,000	55,24
[Ga-68]	1,88	DD(M)	2535,000	40,72
[Ga-68]DMALTO	8,08	DD(M)	251,000	4,03

Fig. S1. Radio-HPLC of [^{68}Ga]Ga-DMALTO

Comparison of μPET/CT evaluation procedures

The evaluation of the activity in the affected muscles was defined manually via μPET/CT. The PET scans were fused with the CT scans. A volume of interest (VOI) was first positioned via the CT image so that the VOI did not reach into the tibia or knee joint. Secondly, the VOI was adjusted via PET images around the fibula or above the fibula when the leg was significantly swollen. Because soft tissue in the hindlimbs is not well represented in CT scans and the mice did not lie at exactly the same position for each scan, it is not possible to match the total and only muscle mass with the positioned VOI. Furthermore, [^{18}F]FDG uptake in the bone marrow of the tibia and knee joints could interfere with the selected VOI. To judge these effects, two independent people analyzed the data. Each evaluator kept the size of the VOI constant for all mice and muscles. Comparison of the evaluation of both people is shown in Fig. S2. The results show that even with a VOI that does not match perfectly the muscle mass, the results of both evaluation processes show no significant difference. Consequently, the basic statement of all experiments is not influenced by the evaluation procedure or person.

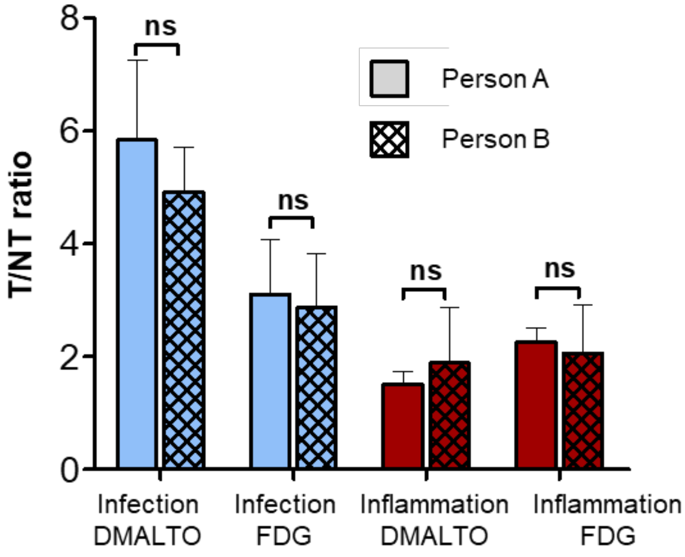

Fig. S2. Comparison of the evaluation procedures. Two independent people (A + B) evaluated for infection or inflammation with [^{68}Ga]Ga-DMALTO or [^{18}F]FDG in both hindlimbs of mice.

GMP-production of [^{68}Ga]Ga-DMALTO

Radiosynthesis was performed on a qualified GAIA GMP automated radiosynthesizer (Elysia-raytest, Straubenhardt, Germany). Operational qualification of the radiosynthesizer, including testing and calibration of the electrical heating and cooling module, radiation detectors, pumps, valve mechanisms, and reactor, is performed periodically.

The process validation of the labeling sequence in Figure S3 was done under GMP-conditions with a quality risk management approach. Briefly, 30 nmol of DMALTO precursor in 2.1 mL 3M sodium acetate buffer and 1 mL ethanol (95%) is transferred into the reaction vessel and warmed to 40 °C. [^{68}Ga]$GaCl_3$ in 1.1 mL 0.1N HCl is eluted from the generator directly into the

reaction vessel, and heated to 100 °C for 12 min. The reaction is quenched by addition of 5 – 8 mL of water. The reaction mixture is passed over a NH2 Sep-Pak plus cartridge (Waters AG, Germany) to remove excess of free [^{68}Ga]$GaCl_3$. The solution is further transferred via a sterile filter into a final product vial.

A

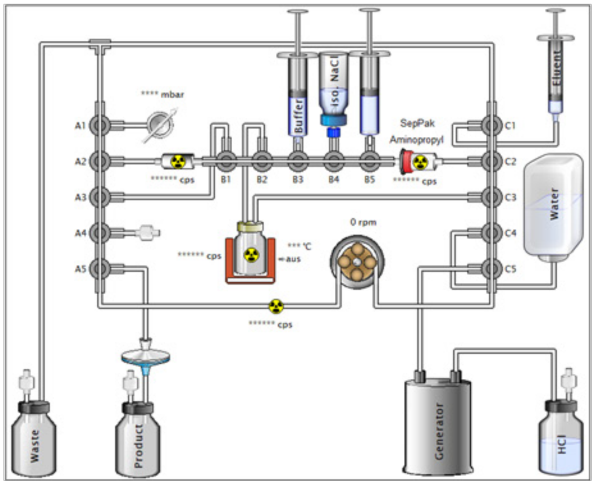

B

	A1	A2	A3	A4	A5	B1	B2	B3	B4	B5	C1	C2	C3	C4	C5	Pump	Heater	Wait Condition	Wait Timeout	Measurement	Description (English)
0	0	0	0	0	0	0	0	0	0	0	0	0	0	0	0	off	off				Initial State
1	0	0	3	1	0	1	0	0	0	0	3	3	0	0	0	250	off	Fixed delay	15		NH2 Purge
2	1	2	2	1	0	2	0	0	0	0	0	3	2	0	0	250	off	Pressure > 2000mbar	180		Kit Integrity Test: Pressurizing
3	1	2	2	0	0	2	0	0	0	0	0	3	3	0	0	off	off	Fixed delay	5		Kit Integrity Test: Equilibration
4	1	2	2	0	0	2	0	0	0	0	0	3	3	0	0	off	off	Fixed delay	15	Gaia–Pressure changes less than 200.0mbar	Kit Integrity Test: Measuring
5	2	2	2	2	0	2	0	0	0	0	0	2	2	0	0	off	off	Fixed delay	5		Kit Integrity Test: Venting
6	0	0	0	1	0	0	0	0	0	0	3	0	0	0	0	100	off	Fixed delay	10		NaCl Syringe Preparation
7	0	0	0	1	0	0	1	3	0	0	0	0	3	0	0	200	off	Fixed delay	15		Buffer Addition: Preparation
8	0	0	0	1	0	0	1	3	0	0	0	0	1	0	0	off	off	Fixed delay	2		Buffer Addition: Preparation Venting
9	0	0	0	1	0	0	1	3	0	0	0	0	3	0	0	-250	off	Fixed delay	30		Buffer Addition
10	1	0	0	2	0	0	0	0	0	0	0	0	3	0	0	off	off	Fixed delay	3		Buffer Addition: Venting
11	1	1	0	0	0	0	0	0	0	0	0	1	0	3	0	-150	off	Fixed delay	30		Cartridge Activation: SCX + NH2
12	1	1	1	0	0	1	0	0	0	0	0	1	0	3	0	-150	off	Fixed delay	10		Cartridge Activation: NH2
13	1	1	3	0	0	1	0	0	0	0	0	0	0	0	0	250	off	Fixed delay	5		Cartridge Activation: Purge Preparation
14	1	0	3	1	0	1	0	0	0	0	0	0	0	0	0	250	off	Fixed delay	5		Cartridge Activation: Purge 1
15	0	0	3	1	0	1	0	0	0	0	3	3	0	0	0	250	40	Fixed delay	10		Cartridge Activation: Purge 2
16	0	3	3	1	0	0	0	0	0	0	3	3	0	0	0	250	40	Fixed delay	10		Cartridge Activation: Purge 3
17	0	2	3	1	0	0	0	0	0	0	3	0	0	0	0	off	40	Fixed delay	3		Cartridge Activation: Venting
18	0	0	0	0	0	3	0	0	0	0	3	0	0	3	3	-20	60	Flow > 1000cps	300		Generator Elution: Waste (1000 cps 300s)
19	0	3	1	0	0	3	0	0	0	0	3	0	0	3	3	-20	90	Fixed delay	120		Generator Elution: Collect
20	0	0	0	0	0	0	0	0	0	0	3	0	0	3	3	-20	90	Fixed delay	10		Generator Elution: Waste
21	0	0	0	0	0	0	0	0	0	0	3	0	0	3	0	-100	100	Fixed delay	10		SCX Washing: Line
22	0	3	1	0	0	3	0	0	0	0	3	0	0	3	0	-100	100	Fixed delay	10		SCX Washing
23	0	0	3	0	0	1	0	0	0	0	3	3	0	0	0	250	100	Fixed delay	5		SCX Washing: Line Purge Preparation
24	0	0	3	1	0	1	0	0	0	0	3	3	0	0	0	250	100	Fixed delay	15		SCX Washing: Line Purge
25	0	3	0	1	0	0	0	0	0	0	3	3	0	0	0	250	100	Fixed delay	10		SCX Washing: Cartridge Purge
26	0	0	0	0	0	0	0	0	0	0	3	0	0	0	0	-70	100	Fixed delay	12	Gaia–Manifold Detector 1	SCX Elution: Preparation
27	0	1	0	0	0	0	3	0	0	0	3	0	0	0	0	-20	100	Fixed delay	15		SCX Elution 1
28	1	1	0	0	0	0	3	0	0	0	3	0	3	0	0	-20	100	Fixed delay	60		SCX Elution 2
29	1	1	0	0	0	0	3	0	0	0	3	0	3	0	0	-250	100	Fixed delay	10		SCX Elution: Purge
30	1	1	0	0	0	3	0	0	0	0	3	0	0	0	0	off	100	Fixed delay	10	Gaia–Reactor Detector	Labeling / Measuring Reactor Activity
31	1	1	0	0	0	3	0	0	0	0	3	0	0	0	0	off	100	Fixed delay	240		Labeling Part 1
32	0	0	0	0	0	3	3	0	0	0	3	0	3	0	0	-40	100	Fixed delay	10		Labeling / Lift Purge
33	0	0	0	0	0	3	0	0	0	0	3	0	0	0	0	off	100	Fixed delay	340		Labeling Part 2
34	0	0	0	0	0	3	1	0	0	3	3	0	3	0	0	150	40	Fixed delay	10		Preparation Trapping
35	0	0	0	1	0	3	1	0	0	3	3	0	3	0	0	250	40	Fixed delay	30		Preparation Trapping 2
36	0	0	0	1	1	3	1	0	0	1	0	3	0	0	0	-80	40	Fixed delay	30		NH2 Trapping: Preparation
37	0	0	0	1	1	3	1	0	0	1	0	3	0	0	0	-150	40	Fixed delay	10		NH2 Trapping
38	0	1	1	0	0	1	3	0	0	0	0	0	1	3	0	-250	40	Fixed delay	8		Washing Reaktor
39	0	1	1	0	0	1	1	0	0	3	0	1	3	0	0	150	40	Fixed delay	5		NH2 Washing Preparation
40	0	1	1	1	0	1	1	0	0	3	0	1	3	0	0	250	40	Fixed delay	30		NH2 Washing
41	0	1	1	0	1	1	0	0	0	1	0	3	0	0	0	-100	off	Fixed delay	20		NH2 Preparation Elution
42	0	1	1	0	1	1	0	0	0	1	0	3	0	0	0	-150	off	Fixed delay	20		NH2 Elution
43	1	0	0	3	1	1	0	0	1	0	0	3	0	0	0	-150	off	Fixed delay	60		Formulation
44	1	0	1	3	1	1	0	0	0	0	0	3	0	0	0	-200	off	Fixed delay	15	Gaia–Manifold Detector 2	Formulation: Purge / Measuring NH2 Post_Elution
45	1	0	1	0	1	1	0	0	0	0	0	3	0	0	0	off	off	Fixed delay	5	Gaia–Manifold Detector 1	Venting Preparation / Measuring SCX Post_Elution
46	1	0	1	0	1	1	0	0	0	0	0	3	0	0	0	off	off	Fixed delay	2	Gaia–Reactor Detector	Measuring Reactor Activity
47	1	0	2	0	0	1	0	0	0	0	0	2	0	0	0	off	off	User prompt	1		Remove Product, Connect Waste
48	0	0	0	0	1	0	0	0	0	0	0	0	0	3	0	-250	off	Fixed delay	30		Filter Integrity Test: Filter Purge
49	1	0	0	0	0	0	0	0	0	0	0	0	0	0	0	250	off	Fixed delay	5		Filter Integrity Test: Line Purge Preparation
50	1	0	0	1	0	0	0	0	0	0	0	0	0	0	0	250	off	Fixed delay	15		Filter Integrity Test: Line Purge 1
51	0	0	3	1	0	1	0	0	0	0	0	3	0	0	0	250	off	Fixed delay	15		Filter Integrity Test: Line Purge 2
52	0	0	3	0	0	1	0	0	0	0	0	3	0	0	0	-250	off	Fixed delay	10		Filter Integrity Test: Line Purge 3
53	1	0	0	0	2	0	0	0	0	0	0	0	0	0	0	-250	off	Pressure > 2500mbar	120		Filter Integrity Test: Pressurizing
54	1	0	0	0	2	0	0	0	0	0	0	0	0	0	0	-60	off	Pressure > 2900mbar	200		Filter Integrity Test: Approaching Bubble Point Value
55	1	0	0	0	2	0	0	0	0	0	0	0	0	0	0	-30	off	Sterile Filter Test	600		Filter Integrity Test: Measuring Bubble Point Value
56	2	0	0	2	2	0	0	0	0	0	0	2	2	0	0	off	off	Fixed delay	5		Venting
57	0	0	0	0	0	0	0	0	0	0	0	0	0	0	0	off	off	Stop Synthesis	5		Stop

Fig. S3. Specific operating parameters of the automated radiosynthesizer

Synthesis of the precursor DOTA-maltohexaose (DMALTO)

To obtain a new radiotracer which, on one hand targets bacteria, and on the other hand is detectable by PET, we used maltohexaose and coupled it to the bifunctional chelator DOTA to allow radiolabeling with [^{68}Ga]Gallium. The synthesis of precursor DOTA-maltohexaose (DMALTO) followed the scheme described in Figure S4. An alkyne-DOTA derivative (3.75 µmol, 1.4 equivalent) was conjugated to azide-functionalized maltohexaose (2.67 µmol, 1 equivalent) using Cu(I)-catalyzed alkyne-azide 1,3-dipolar (click) cycloaddition (CuAAC).

The catalytic amounts of Cu(I) typically used for CuAAC reactions could not be applied because the DOTA moiety preferentially complexed copper ions [Bock VD, Hiemstra H, van Maarseveen JH. CuI-Catalyzed Alkyne–Azide "Click" Cycloadditions from a Mechanistic and Synthetic Perspective. Eur J Org Chem. 2006; 2006(1): 51–68; Struthers H, Mindt TL, Schibli R. Metal chelating systems synthesized using the copper(I) catalyzed azide-alkyne cycloaddition. Dalton transactions (Cambridge, England: 2003). 2010; 39(3): 675–696; Meldal M, Tornoe CW. Cu-Catalyzed Azide - Alkyne Cycloaddition. Chem Rev. 2008; 108: 2952–3015]. As a result, catalytic quantities of Cu(I) were sequestered by DOTA and were unavailable for the cycloaddition reaction. Only upon addition of a stoichiometric excess of copper (2.8 equivalents) was a rapid and quantitative reaction achieved. Subsequently, copper complexed to the DOTA moiety was removed by precipitation using a 31-fold molar excess of sodium sulfide, enabling quantitative copper removal (CuS solubility product: Ksp = $6 \cdot 10^{37}$). In a third step, acid-mediated deprotection was performed to obtain the final precursor, DMALTO. Following purification, DMALTO was analyzed by electrospray ionization mass spectrometry (ESI-MS),

which confirmed the absence of copper–DMALTO complexes. This is a critical prerequisite for efficient [^{68}Ga]Gallium radiolabeling and for meeting precursor purity requirements under GMP conditions.

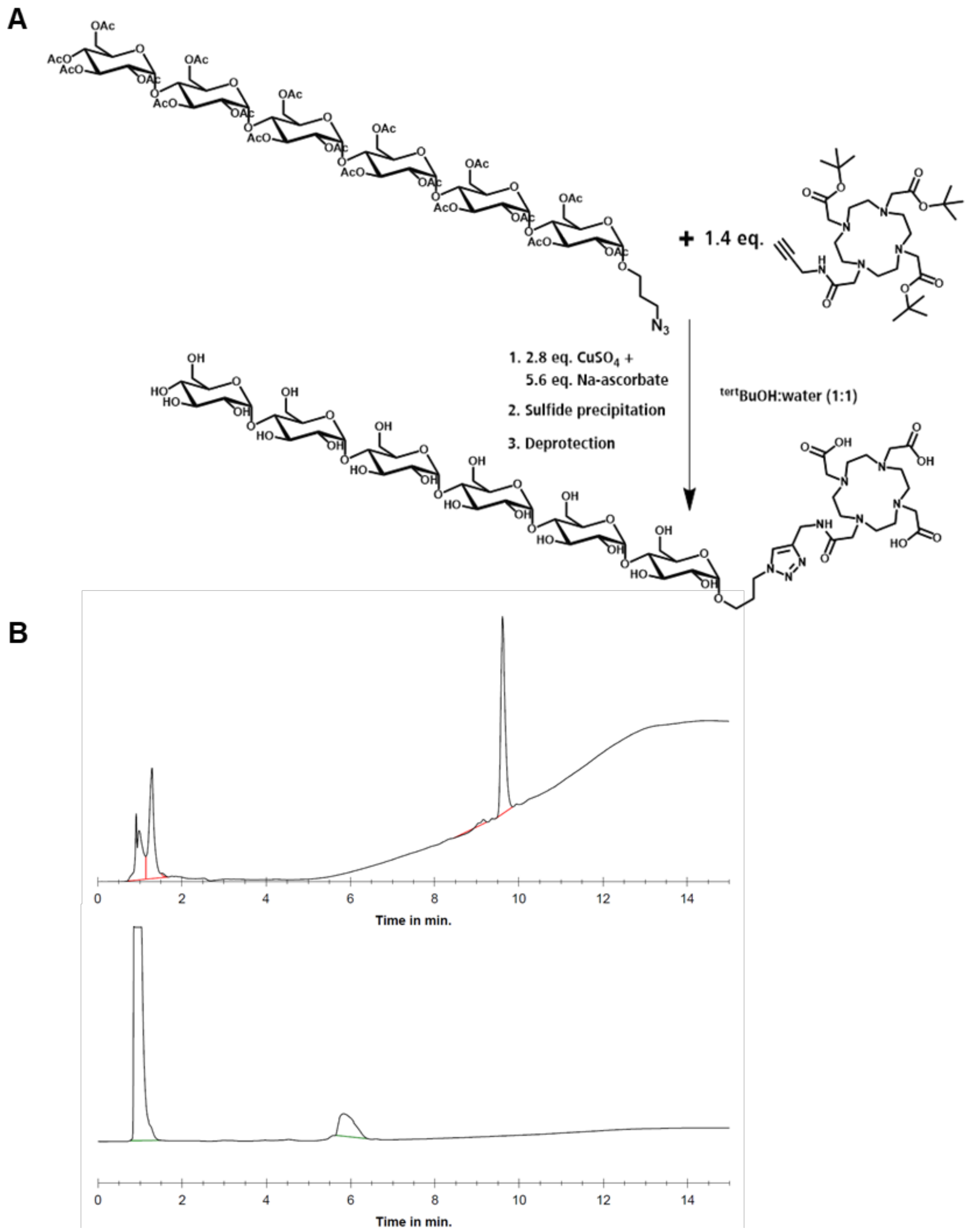

Fig. S4. Synthesis of precursor DMALTO. (**A**) Synthesis scheme of DMALTO. (**B**) Analytical HPLC chromatogram at the beginning (top) and end (bottom) of reaction: quantitative conversion of maltohexaose (9.5 min).

Imaging aseptic inflammation with [^{18}F]FDG over time

To the best of our knowledge, the time point when inflammation peaks after LPS injection into the hindlimb muscle has not been described. Therefore, we chose to investigate inflammation using [^{18}F]FDG PET imaging. We evaluated the uptake of [^{18}F]FDG over time between two hours and seven days after injection of LPS. Fig. S5 indicates that the inflammation peaked at day 3.

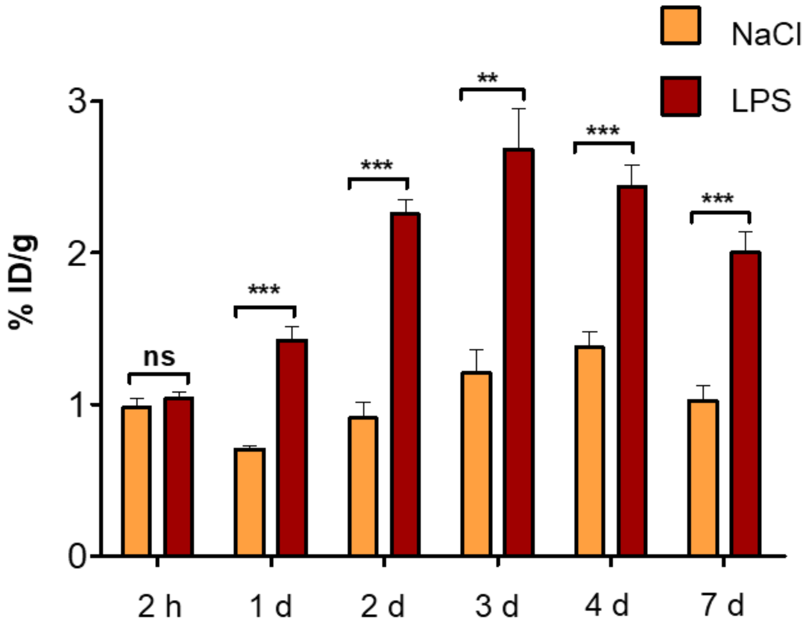

Fig. S5. Aseptic inflammation over time. Relative activity accumulations in %ID/g unit of [^{18}F]FDG at different time points (2h – 7d).
